# Building sustainable SBIRT in an integrated hospital system in New York

**DOI:** 10.1186/1940-0640-10-S1-A44

**Published:** 2015-02-20

**Authors:** Megan A O'Grady, Sandeep Kapoor, Mark Auerbach, Jeanne Morley, John Yu, Veronica Salvas, Charles Neighbors, Jon Morgenstern, Joe Conigliaro, Nancy Kwon, Tom McGinn, John Kane

**Affiliations:** 1 Department of Health Services Research, National Center on Addiction and Substance Abuse at Columbia University (CASAColumbia), New York, NY, 10017, USA; 2 North Shore-Long Island Jewish Health System, New Hyde Park, NY, 11040, USA; 3 New York State Office of Alcoholism and Substance Abuse Services (OASAS), Albany, NY, 12203-3526, USA

## 

Screening, Brief Intervention, and Referral to Treatment (SBIRT) for substance use has received a great deal of empirical support. Despite the evidence for effectiveness of SBIRT and a compelling rationale for its integration into health-care settings, the circumstances under which it is likely to be implemented and sustained remain elusive. In this project, SBIRT was implemented into emergency departments (EDs) and primary care practices (PCPs) within a large, integrated health system in New York serving areas heavily affected by Hurricane Sandy. The SBIRT model was implemented in the following way: 1) Front-line staff (e.g., nurses and medical office assistants) administered a five-item SBIRT prescreen for alcohol, drug, and tobacco use to all patients during triage; 2) If a positive prescreen resulted, a health educator conducted a full screen using the AUDIT and/or DAST-10; and 3) The health educator conducted a brief intervention and referral to addiction treatment as indicated by the full-screen score. To assist in building the SBIRT process into the normal site workflow, prescreening questions were integrated into the electronic medical record (EMR) in a user-friendly way for front-line staff to administer. The EMR automatically scored the prescreen. As of May 2014, SBIRT was implemented in three EDs and two PCPs within the health system, with services starting on 12/1/13. Implementation was evaluated by reviewing EMR data as well as patient data collected at each site. Approximately 13000 patients were prescreened (13% positive). More women than men were prescreened (61% female), and there was a significant older population (9% were 18–24; 39% were 55 or older), as well as diversity in ethnicity (24% Latino) and race (19% African American). Almost 900 full screens were conducted (50% positive), which resulted in 284 brief interventions and 149 referrals to brief or formal addiction treatment. Further analysis revealed that multiple substances were an issue for many patients. For example, 20 percent of patients were positive on both the AUDIT and the DAST-10, and 39 percent of patients who full-screened positive for drugs or alcohol also screened positive for past-year tobacco use. Severity of full-screening scores was greater in the ED setting as compared to the PCP setting. Thirty-nine percent of patients who full-screened positive reported marijuana use in the past 30 days, and 14 percent were daily marijuana users. Overall, results suggest that SBIRT was successfully implemented in several busy sites within this large health system.

